# Impacts of Climate Change on Alpha and Beta Diversity Patterns of Cerrado anurans

**DOI:** 10.1002/ece3.73983

**Published:** 2026-08-25

**Authors:** Amanda Sabino, Gabriela Alves‐Ferreira, Neander Marcel Heming, Gastón Andrés Fernandez Giné, Daniela Custódio Talora

**Affiliations:** ^1^ Applied Ecology and Conservation Lab, Programa de Pós‐graduação em Ecologia e Conservação da Biodiversidade Universidade Estadual de Santa Cruz Ilhéus Brazil; ^2^ Laboratório de Análise e Síntese em Biodiversidade Universidade Estadual Paulista “Júlio de Mesquita Filho” Rio Claro Brazil

**Keywords:** Anura, community structure, global warming, species distribution, species distribution model, suitable area

## Abstract

Climate change poses a major threat to biodiversity, altering species distributions and reshaping ecological communities. In the Brazilian Cerrado, a highly threatened tropical savanna, future climate projections indicate increased temperatures and reduced precipitation, potentially driving significant shifts in anuran diversity. Here, we assess how climate change (2050, SSP585) affects alpha and beta diversity of 139 anuran species in the Cerrado and explore their relationships with two key climate dimensions: climate anomaly and climate change velocity. We used occurrence records and bioclimatic variables to build Species Distribution Models (SDMs) via Maxent, and using the binary maps generated by the SDMs, we calculated alpha and beta diversity and used spatially explicit regressions to investigate the associations of diversity metrics with climatic metrics. Our results indicate a strong reduction in species richness across most of the biome, with the southeast and southwest experiencing the highest losses of up to 40 species. Conversely, northern and northeastern regions show richness gains. Changes in community composition were mainly driven by species replacement, particularly in the northwest, west, and east. No significant relationship was detected between climatic anomalies and changes in species richness. Instead, higher velocity in the change of temperature extremes was associated with richness losses, while higher precipitation change velocity was linked to richness gains. Maximum temperature anomalies and precipitation change velocity were key predictors of community restructuring. These findings highlight the urgent need for regional conservation planning to mitigate biodiversity loss driven by climate change in the Cerrado.

## Graphical abstract

We modeled the distribution of 139 anuran species to assess how climate change will reshape biodiversity patterns in the Brazilian Cerrado. Our results predict widespread species loss and reveal that biotic communities will be primarily restructured through species replacement.

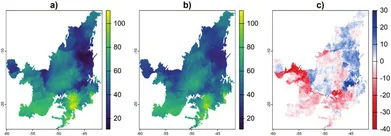

## 1 Introduction

Climate is one of the main determining factors of species distribution and richness patterns (Lenoir and Svenning 2015; Liu et al. 2017; Arneth et al. 2020). Anthropogenic climate change, however, is forcing rapid rearrangements in biodiversity as suitable habitats expand, contract, or shift (Li et al. 2009; Lenoir and Svenning 2015; Hannah et al. 2020), leading not only to loss of species locally but also to the redistribution and establishment of novel assemblages, with direct consequences for diversity patterns (Ochoa‐Ochoa et al. 2019; Wudu et al. 2023).

For amphibians, these shifts are particularly critical due to their sensitivity to environmental changes (Alan Pounds et al. 2006; Blaustein et al. 2010). In the Brazilian Cerrado, a global biodiversity hotspot and an extremely threatened savannah, future projections indicate it will become hotter and drier by 2050 (Hofmann et al. 2021). The biome is under intense agricultural pressure and is also expected to experience high future climatic instability (Borges et al. 2019), potentially reducing species' ability to track shifting climatic conditions (Wudu et al. 2023).

While standard bioclimatic variables (e.g., mean temperature and precipitation) are widely used to project future distributions, they often fail to capture the multi‐dimensional nature of climate change. Metrics such as climate anomaly (the magnitude of change) and climate change velocity (the rate of climatic dislocation across space) can provide greater insight into extinction risks and dispersal requirements (Coumou and Rahmstorf 2012; Garcia, Cabeza, et al. 2014; Harris et al. 2020; Thakur et al. 2022).

Despite recent advances in modeling Cerrado anurans (e.g., Vasconcelos et al. 2018; Alves‐Ferreira et al. 2022), most studies have focused primarily on species richness or broad distributional shifts. Consequently, the role of climate velocity and extreme anomalies in driving changes in both alpha and beta diversity remains unexplored. Furthermore, partitioning beta diversity into replacement and richness difference components is important to distinguish whether communities are being replaced or simply impoverished (Baselga 2010, 2012; Cardoso et al. 2015). Here, we evaluated the effects of climate change on the diversity of 139 Cerrado anuran species. Specifically, we aimed to: (I) evaluate the impact of climate change on alpha (hereafter referred to as species richness) and beta diversity, identifying regions of species loss, gain, as well as composition changes; and (II) evaluate if projected changes in richness and beta diversity are associated with climate anomaly and climate velocity across the Cerrado. We expected that increases in temperature and drier conditions would lead to a contraction of climatically suitable area for species, leading to reductions in anuran richness in most of the Cerrado and significant changes in species composition. We also predicted that regions with faster climate velocity and high anomaly, particularly for temperature extremes, would experience the highest richness losses and greater composition shifts.

## 2 Methods

### 2.1 Study Area

The Cerrado biome, covering approximately 23% of Brazil's land area and eight states of Central Brazil, is the largest savanna in the world and the second largest biome in the country. Recognized as a biodiversity hotspot (Myers et al. 2000), the Cerrado shows a variation in its phytophysiognomies, ranging from open grasslands to dry forests (Hunke et al. 2015). However, as the most threatened savanna globally, the Cerrado faces high future climate instability (Borges et al. 2019; Borges and Loyola 2020), with projections indicating hotter and drier conditions (Hofmann et al. 2021) that pose risks to its biodiversity.

### 2.2 Occurrence Data

Anurans have the highest level of endemism among vertebrates in the Cerrado region (Klink and Machado 2005; Silvano and Segalla 2005). We analyzed a total of 139 anuran species from the Cerrado biome, representing a diverse taxonomic distribution across 15 amphibian families. The most represented families in our dataset were Hylidae (61 species), Leptodactylidae (37 species), and Bufonidae (13 species), which together accounted for the majority of the species considered (Online Resource 1). We obtained the occurrence data for 139 anuran species from scientific literature (data provided by Vasconcelos et al. 2018) and online database Global Biodiversity Information Facility (GBIF.org 2024). We removed duplicates, incomplete and unlikely coordinates using the *scrubr* package (v0.4.0; Chamberlain 2021). In order to reduce spatial autocorrelation and possible geographic biases, we removed records that were within 10 km of each other using the *spThin* (v0.2.0) optimization algorithm (Aiello‐Lammens et al. 2015), implemented in the R programming language (v4.3.2; R Core Team 2023). This procedure helps to reduce sampling bias and improves model performance (Boria et al. 2014; Radosavljevic and Anderson 2013). Given the heterogeneity of the biome, the 10 km cut‐off threshold allowed us to reduce sampling bias without decreasing the number of climatically distinct locations available for modeling (Anderson and Raza 2010). In total, 15,601 occurrence records were maintained in the database (Online Resource 2).

We considered only species with more than five records, a widely adopted threshold for species distribution modeling (SDM) studies, which allows for greater inclusivity while maintaining model robustness (Pearson et al. 2007; Van Proosdij et al. 2016; Nneji et al. 2023). This criterion enhances the inclusivity and representativeness, considering rare and endemic species with restricted distributions. It also ensures model robustness and helps prevent statistical issues associated with extremely small sample sizes. The algorithm used for modeling, Maxent, performs well and accurately even with relatively small sample sizes (Hernandez et al. 2006).

### 2.3 Environmental Variables

We downloaded a set of 19 bioclimatic variables from the WorldClim v2.1 platform (Fick and Hijmans 2017), with a spatial resolution of 2.5 arc minutes (~4.5 km^2^ at the equator), for the baseline (1970–2000) and future scenarios (2041–2060). We considered three global circulation models (AOGCMs: CNRM‐CM6‐1, MIROC6, and MRI‐ESM2‐0) to maximize variability by selecting models from different clusters (Varela et al. 2015). Additionally, we included two shared socio‐economic pathway scenarios, one pessimistic (SSP585) and one optimistic (SSP245). However, while both were used for initial modeling, we present the results only for the pessimistic scenario (SSP585) as preliminary analyses indicated qualitatively similar spatial patterns for both pathways. After defining the calibration area for each species (see method below), we estimated Pearson's correlation coefficient between the variables from the baseline using the “select_vars_b” function from the *ENMwizard* package (v0.4.2; Heming et al. 2018). To reduce collinearity problems, we only selected variables (those with greater biological relevance) with Pearson's correlation <|0.7| to perform the SDMs (Online Resource 3).

Additionally, we downloaded monthly weather data for average minimum, maximum temperature (°C) and total precipitation (mm) for 1981–2000 and for 2041–2060 (SSP 585) from the WorldClim v2.1 database at the resolution of 2.5 arcmin (Fick and Hijmans 2017). To quantify the magnitude of local climatic anomalies, we calculated the sum of Standardized Euclidean Distances (SED, Garcia, Cabeza, et al. 2014) using the “ccm” function from the *climetrics* package (Taheri et al. 2023). We calculated the SED for precipitation and maximum temperature individually, between the years 1981–2000 (baseline scenario) and 2041–2060 (future scenario). High anomaly values indicate greater local climate change (Williams et al. 2007). We also calculated the distance‐based climate change velocity (Garcia, Cabeza, et al. 2014) for the two variables mentioned above using the function “ccm” from the package *climetrics* (v1.0‐15; Taheri et al. 2023) (see details on Online Resource 4).

### 2.4 Species Distribution Models (SDMs)

We used the filtered occurrence data and the selected bioclimatic variables to predict climatically suitable areas for 139 anurans in the Cerrado. We followed the ODMAP (Overview, Data, Model, Assessment, and Prediction) protocol (Zurell et al. 2020) to make the workflow and parametrization of our models reproducible (see details in Online Resource 5). We built SDMs through the MaxEnt algorithm (Phillips et al. 2017) using the *ENMwizard* package (v0.4.2; Heming et al. 2018) from the R programming language (v4.3.2; R Core Team 2023). We defined the calibration area for model fitting through a 1.5° buffer around a Minimum Convex Polygon (MCP) involving all filtered occurrence points for each species (Anderson and Raza 2010; Barve et al. 2011; Heming et al. 2018). To apply a model selection structure, we build 70 models for each species, considering all the possible combinations of Feature classes (FCs): linear (L), product (P), quadratic (Q), hinge (H) and Regularization multipliers (RMs) with values ranging between 0.5 and 5, with intervals of 0.5. For validation and evaluation of the predictive ability of the models, we applied two methods of geographic partitioning of occurrence records: ‘block’ and ‘jackknife’ using the ENMevaluate_b function of the *ENMwizard* package (v0.4.2; Heming et al. 2018). Block cross‐validation partitions the occurrence records into different blocks for model evaluation and calibration (Roberts et al. 2017), while the jackknife method removes only one record at a time and builds a new model in the absence of that record (Pearson 2010; Shcheglovitova and Anderson 2013). We used the jackknife method for species with fewer than 15 occurrence records, while the block method was used for species with more than 15 records. We calibrated and evaluated the models with the current baseline climate scenario (see details of species‐level model performance summary on Online Resource 6).

After this procedure, we selected 10% of the best performing models, which were chosen based on the lowest omission rate (OR) values and the highest area under the curve (AUC) averages, following the sequential procedure suggested by Boria et al. (2017). We projected the selected models for the baseline and future scenarios, considering the year 2050, one pessimistic shared socioeconomic pathway (SSP585). We calculated the average to generate a consensus model for each species and each scenario, combining the projections resulting from the three GCMs. Finally, the continuous suitability maps were converted into binary maps (1 = presence and 0 = absence) using the 10 percentile training presence (10ptp) threshold, since the threshold value should be adjusted to the prevalence of presence data (Lobo et al. 2008) and it assumes that 10% of records, with smaller values of environmental suitability, will be discarded (Liu et al. 2005; Barros et al. 2012). To visualize the spatial patterns of the models, we provide species distribution maps overlaid with occurrence records (Online Resource 7).

### 2.5 Diversity Metrics

For the community diversity patterns, we calculated the alpha and beta diversity considering only the baseline and pessimistic scenario because preliminary analyses indicated that the results are very similar between the optimistic and pessimistic scenarios. We estimated the spatial pattern of alpha diversity (species richness) by summing the binary maps of species for each scenario (baseline and future) using the “rast.sr” function from the R package *phyloraster* (v2.3.0; Alves‐Ferreira et al. 2024). Then, we estimated the temporal change in species richness (delta richness), calculating the difference between the richness in the baseline and in the future scenario using the “delta.grid” function from *phyloraster* v2.3.0 (Alves‐Ferreira et al. 2024). Positive values of “delta richness” indicate species gain in the future, while negative values indicate species loss.

In addition, we calculated temporal beta diversity between the baseline and the future scenarios (SSP585) using the function “temp.beta” from the *divraster* package (v1.2.3; Mota et al. 2023). We partitioned beta diversity into replacement and richness difference components (Cardoso et al. 2015) to evaluate which component was predominant in determining the beta diversity. Values of beta ratio close to one represent the predominance of species replacement and values close to zero indicate predominance of gain/loss of species (richness difference component).

### 2.6 Analysis

We calculated the Variance Inflation Factor (VIFs) using the function ‘vif’ from package *car* (v3.1‐5; Fox and Weisberg 2019) to evaluate if there was multicollinearity between the predictors variables (Precipitation anomaly, maximum temperature anomaly, minimum temperature anomaly, velocity of change in precipitation, maximum and minimum temperature), but variables were not strongly related (VIFs < 3). To evaluate the relationship between community metrics (delta richness and beta diversity) and climatic predictors, we fitted models using the *sdmTMB* package (v1.0.0; Anderson et al. 2022) to account for the spatial structure of the data. First, we built a spatial mesh based on the spatial coordinates of the variables with a cutoff of 1. Models were fitted for each response variable considering all climatic predictors. In the model for beta diversity, we included the baseline species richness as a predictor variable to control for the effect of species already present in each cell at the raster, and in both models we also included elevation to help reduce spatial autocorrelation and control their effects, downloaded from the WorldClim v2.1 database at the resolution of 2.5 arcmin (Fick and Hijmans 2017). For each response variable, we tested different error distributions, including Beta, Gamma, Gaussian, and Log‐normal, selecting the best model based on the Akaike Information Criterion (AIC). For beta diversity, the best model was the Beta distribution, while for delta richness, we selected the Gaussian distribution. All analyses were performed in the R programming language (v4.3.2; R Core Team 2023).

## 3 Results

**FIGURE 1:**
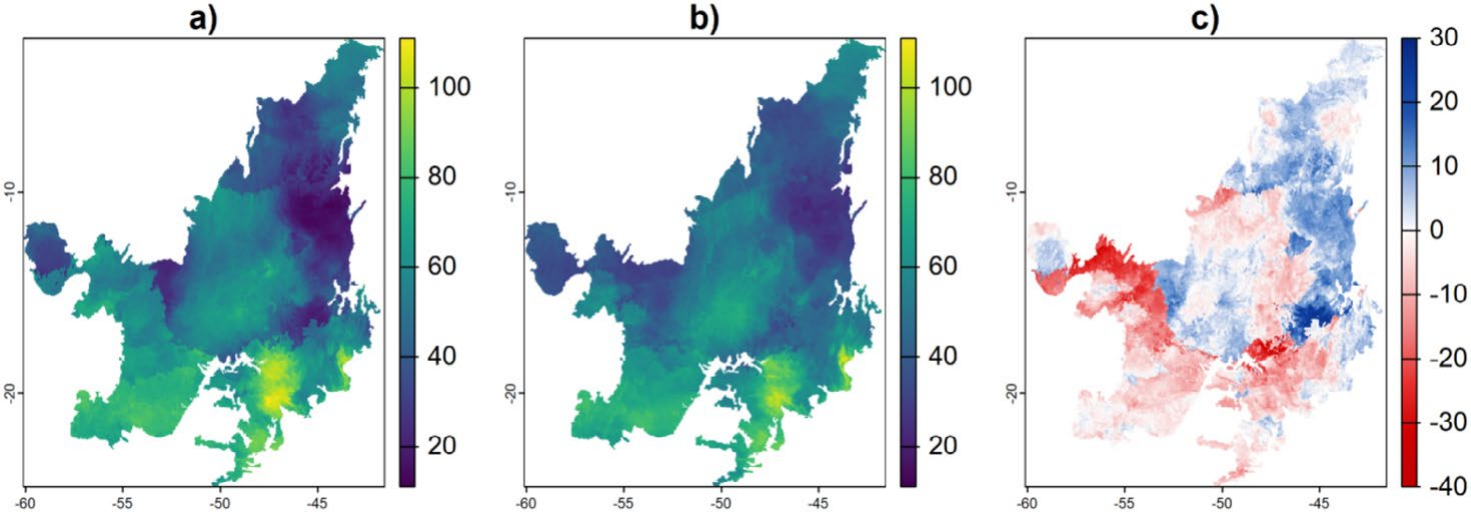
Spatial patterns of anurans species richness in the Brazilian Cerrado for (a) the current scenario (baseline), (b) pessimistic future climate scenarios (year 2050, SSP585), and (c) difference between the future and current richness (delta richness). In panel (a) and (b), red, orange, and yellow colors represent regions with high species richness, while green, dark blue, and light blue represent regions with low species richness. In panel (c), Red colors represent species losses in species richness, gray/white colors represent regions where species richness is not predicted to change, and blue colors represent gain in species richness.

Our study showed that anuran species richness (alpha diversity) is projected to be currently higher in the southern portion of Cerrado (Figure 1a), while the northeast region presented lower species richness. Our results indicated that most of the Cerrado is predicted to lose species richness in the future (Figure 1b). The greatest richness losses are predicted for the southeast and southwest regions that currently encompass the highest values of species richness, with losses ranging from 30 to 40 species (Figure 1c). Gains in species richness are predicted for the regions north, northeast, and for a small portion of the eastern Cerrado, where a marked richness gain (> 20 species) was predicted (Figure 1c). The anuran species richness ranges from 11 to 111 species in the baseline scenario and from 19 to 108 species in future scenarios.

**FIGURE 2:**
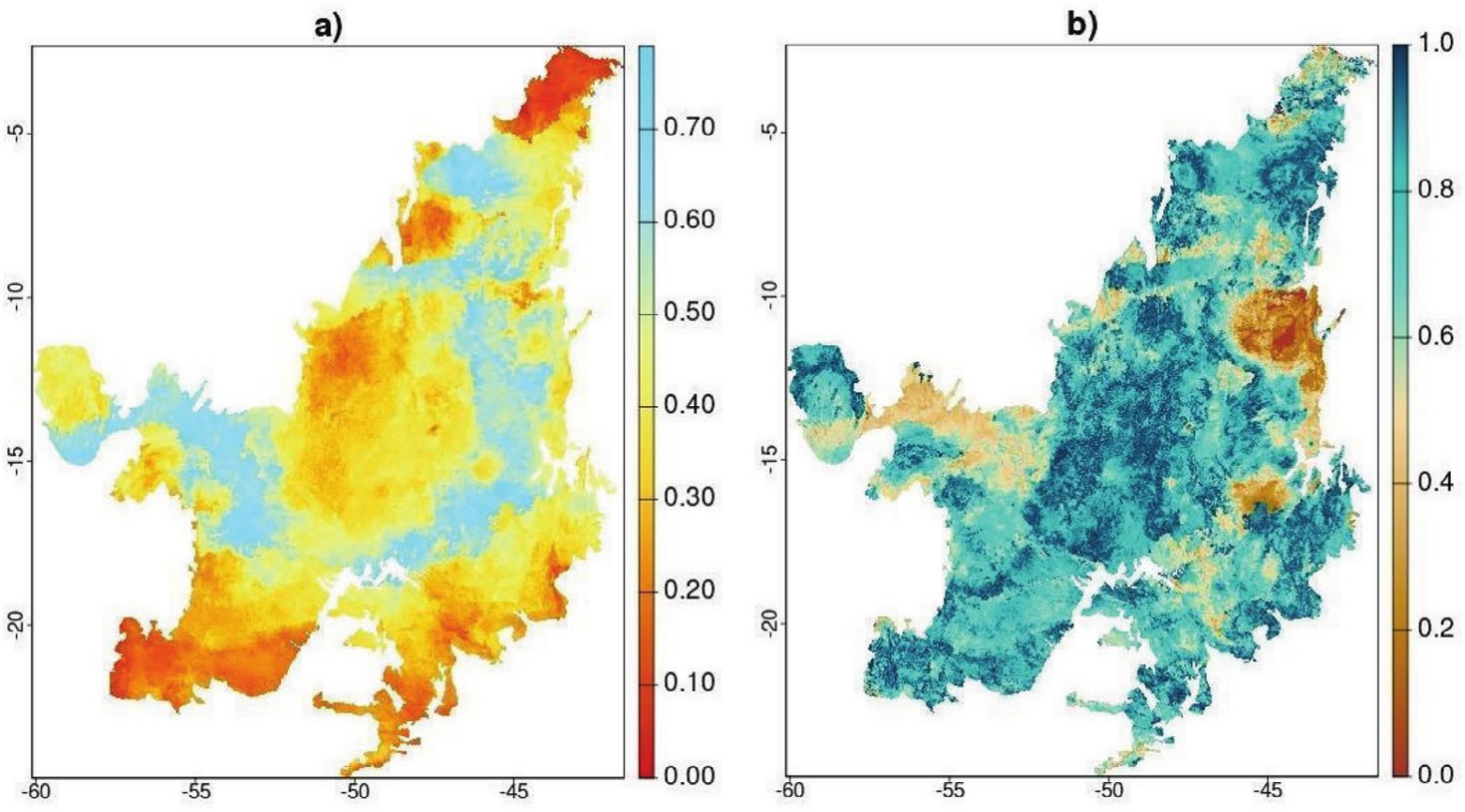
Spatial temporal patterns of anurans beta diversity in the Brazilian Cerrado (a) between the current baseline and future climate scenario, and its (b) components: The proportion of replacement and richness difference. In panel (b) blue color represents the predominance of replacement while brown color indicates predominance of richness difference.

The largest changes in the composition of anurans (beta diversity) were predicted for the northwest, west, and east portion of the biome (Figure 2a), while low to intermediate beta diversity is expected for the north, southwest, and south of the Cerrado (Figure 2a). The partitioning of beta diversity in two components (replacement and richness difference) reveal that the community is projected to be shaped predominantly by the replacement of anuran species in most of the biome (Figure 2b). Contrarily, the richness difference component is observed for a small portion of east center (values close to zero, Figure 2b), where high gains of species richness were predicted (Figure 1c), as also, in small portions of the southeast and southwest regions, where severe losses of species richness were predicted.

The regression analyses (Table 1) revealed that anomalies were not related to delta richness. On the other hand, we observed a negative relationship between the velocity of change in temperature extremes (minimum and maximum) and the delta richness (Table 1), indicating that areas with higher velocity of change a greater loss of species richness or a smaller gain in species was predicted (lower delta values). Inversely, we observed a positive relationship between the precipitation change velocity and delta richness.

**TABLE 1:** Standardized regression coefficients from spatial generalized linear mixed effects models for beta diversity and delta richness of anurans considering the baseline and future scenarios.

Predictors	Estimate	Std. Error	95% CI (Lower)	95% CI (Upper)
**Delta richness (AIC = 133025.1; Pseudo‐*R* ^2^ = 0.81)**				
(Intercept)	−1.4892	1.7092	−4.8391	1.8607
Precipitation Anomaly	6.8702	6.9238	−6.7002	20.4407
Tmax Anomaly	−0.2913	0.4754	−1.2231	0.6405
Tmin Anomaly	1.1390	0.5894	−0.0161	2.2942
Precipitation Change velocity	0.6230	0.2462	0.1404	1.1056
Tmax Change velocity	−0.0625	0.0127	−0.0874	−0.0376
Tmin Change velocity	−0.0275	0.0127	−0.0523	−0.0027
Elevation	−0.0031	0.0003	−0.0037	−0.0025
**Beta diversity (AIC = −78712.4; Pseudo‐*R* ^2^ = 0.90)**				
(Intercept)	−0.4801	0.2274	−0.9258	−0.0344
Precipitation Anomaly	−2.2801	0.3731	−3.0114	−1.5487
Tmax Anomaly	0.4198	0.0257	0.3695	0.4701
Tmin Anomaly	−0.3518	0.0337	−0.4179	−0.2857
Precipitation Change velocity	0.0484	0.0141	0.0206	0.0761
Tmax Change velocity	−0.0027	0.0007	−0.0041	−0.0014
Tmin Change velocity	−0.0020	0.0007	−0.0034	−0.0007
Richness – Baseline	−0.0121	0.0003	−0.0127	−0.0115
Elevation	0.0007	0.0000	0.0006	0.0007

We also evaluated the relationship of climatic predictors on beta diversity. We found that the precipitation and the minimum temperature anomalies were negatively related with beta diversity (Table 1). On the other hand, the anomaly in maximum temperature had a significant and positive relationship with the beta diversity (Table 1). This result indicates that as the anomaly in maximum temperature increases, the greater will be the change in species composition in the future. The velocity of changes in the temperature extremes (minimum and maximum) was negatively related to beta diversity, while the relationship with precipitation change velocity was positive. This result indicates that where the velocity in precipitation changes is higher, greater will be the change in species composition in the future.

The most important variables in predicting range areas for Cerrado anurans were the Precipitation of Warmest Quarter (BIO 18), Isothermality (BIO2/BIO7) (×100) (BIO 3), and Mean Diurnal Range (Mean of monthly (max temp—min temp)) (BIO 2) (Online Resource 3).

## 4 Discussion

Our study predicted a decline in species richness of Cerrado anurans for the year 2050 concentrated in southeast and southwest of the Cerrado with a loss of up to 30 species, while for the northern and northeastern region, the models predicted an increase (gain of up to 20 species) in anuran species richness. Additionally, we showed substantial changes in community composition for the future, primarily driven by species replacement in most of the biome. Our study also demonstrated that changes in anuran richness and beta diversity in the Cerrado are projected to be driven by climatic predictors.

Changes in climate at the regional level can potentially result in either negative or positive consequences for the size and position of species distribution (Garcia, Araújo, et al. 2014; Warren et al. 2018). Our study provides information regarding species loss and restructuring of anuran communities in Cerrado, indicating a significant decrease in species richness across most of the biome, along with a restructuring of anuran communities. This pattern of loss in species richness is congruent with other studies carried out in Cerrado. Vasconcelos et al. (2018) projected widespread richness declines with gains restricted mainly to the northeastern Cerrado, whereas Alves‐Ferreira et al. (2022) showed that most anuran species are expected to lose climatically suitable area, particularly in higher‐altitude regions.

The restructuring of communities and the associated loss of species may compromise ecosystem functioning by disrupting species interactions and trophic dynamics (Rogers and McCarty 2000; Tylianakis et al. 2008; IPBEs 2019). Amphibians play important ecological roles as both predators and prey, contributing to insect population control and nutrient cycling; thus, projected declines in anuran populations may trigger cascading effects on Cerrado ecosystems and reduce ecological stability (Whiles et al. 2006; Hopkins 2007; Wudu et al. 2023).

The southern Cerrado is known to be the region with the highest anuran species richness (Brandão et al. 2004). Despite the high richness, this region exhibits the highest deforestation rates in the entire Cerrado (Strassburg et al. 2017) and the lowest coverage by protected areas (Françoso et al. 2020). Species endemic to the Cerrado may face particularly high risks, as their restricted distribution limits their ability to track shifting climatic conditions (Nori et al. 2016). Additionally, habitat fragmentation caused by deforestation can further isolate populations, reducing gene flow and increasing endogamy, consequently decreasing genetic diversity and adaptive potential in the face of climate change (Carnaval et al. 2009; Wudu et al. 2023). Therefore, in addition to the expected impact of climate change, the anuran community is also affected by the high rates of deforestation, as habitat loss may further constrain species' ability to track shifting climatic conditions (Hof, Levinsky, et al. 2011; Hof, Araújo, et al. 2011; Luedtke et al. 2023).

Ecological traits such as dispersal capacity and niche breadth can contribute to the spatial variation in community composition and can promote species replacement (Dobrovolski et al. 2012). In our study, replacement was the dominant component of beta diversity across most of the Cerrado, indicating widespread substitution of local anuran assemblages by 2050. This result is reinforced by the predicted richness gains in northern and northeastern portions of the biome, which likely reflect species redistribution rather than biodiversity improvement (Batllori et al. 2020; Wudu et al. 2023). Under climate change, species with restricted distributions are more prone to range contractions (Pacifici et al. 2015; Saupe et al. 2015), whereas widespread or generalist species may be more likely to establish in newly suitable areas (Garcia, Araújo, et al. 2014). Such replacement dynamics may lead to increasing biotic homogenization across the biome (Lenoir and Svenning 2015; Socolar et al. 2016).

Our results indicate that distinct climatic dimensions are associated with different aspects of community change. The velocity of temperature extremes was negatively related to changes in species richness, suggesting that areas experiencing faster climatic displacement are more likely to undergo richness loss or smaller richness gains (Loarie et al. 2009; Brito‐Morales et al. 2020). Because climate velocity reflects the rate at which suitable climatic conditions shift across space (Brito‐Morales et al. 2020; Chan et al. 2024), faster velocities may be particularly problematic in fragmented landscapes such as the Cerrado, where dispersal may be limited (Luedtke et al. 2023; Oliveira et al. 2026). In contrast, maximum temperature anomaly showed a positive relationship with beta diversity, indicating that local climatic departures may be more strongly linked to future composition reorganization than to richness change itself (Wudu et al. 2023; Brown et al. 2024). Amphibians generally exhibit narrow thermal tolerance limits, and exposure to extreme temperatures can reduce physiological performance, activity, and local persistence (Ficetola and Maiorano 2016).

Changes in precipitation dynamics may also be particularly important. In our models, richness change and beta diversity was positively associated with precipitation velocity, indicating that shifts in water availability may structure future redistribution patterns. This is especially relevant for anurans, whose reproductive activity, larval development, and seasonal dynamics are tightly linked to precipitation regimes (Araújo et al. 2006; Otani 2025). Future warming scenarios are expected to intensify drought frequency and water stress (IPCC 2022), and the Cerrado is projected to become progressively hotter and drier over coming decades (Hofmann et al. 2021). From a conservation perspective, climate anomaly and climate velocity may imply distinct management priorities: areas with high climatic anomaly may require strategies focused on local persistence, whereas areas with high climate velocity may require conservation actions aimed at maintaining habitat connectivity and dispersal corridors, thereby facilitating species tracking suitable habitats under non‐analogous climatic conditions (Loarie et al. 2009; Bonebrake et al. 2018; Hannah et al. 2020; Arneth et al. 2020).

Niche models are a useful tool to evaluate the effects of climate change on species distributions. However, these models do not explicitly incorporate population‐level mechanisms and processes (e.g., biotic interactions, dispersal, and adaptive potential to abiotic conditions), which implies limitations in the interpretation of model results. The effects of climate change on organisms depend on species' abilities to cope with different environmental challenges and their capacity for adaptation (Chevin et al. 2010), which are not directly considered in ecological niche models. Another limitation is the selection of climatic variables, which may not fully capture the complexity of environmental drivers shaping biodiversity patterns (Elith and Leathwick 2009; Santini et al. 2021). Although climate‐change metrics can be used as proxies for impacts on species distributions, these impacts do not act in isolation (Wudu et al. 2023). They interact with other human‐induced threats, such as human‐dominated areas, which can act as obstacles to species dispersal and establishment, hindering their ability to reach climatically suitable areas (Brook et al. 2008; Warren et al. 2018; Hu et al. 2021). A third limitation is the use of occurrence data from GBIF, which is subject to sampling biases, as regions with limited survey effort or accessibility may be underrepresented (Beck et al. 2014). To mitigate this issue, we applied data‐cleaning procedures to remove duplicate records, ensure taxonomic consistency, and exclude geographically imprecise or erroneous entries (Loiselle et al. 2008; Zizka et al. 2019). Despite these limitations, our study integrates robust modeling frameworks and complementary metrics to enhance the reliability of the findings. Overall, although these limitations should be considered, they do not undermine the main conclusions of the study.

Our results reveal that future climate change will drive substantial declines in anuran species richness across most of the Cerrado, with the greatest losses projected for the southeast and southwest, alongside a widespread restructuring of community composition predominantly driven by species replacement. These changes were associated with distinct climatic dimensions, where the velocity of temperature extremes and precipitation was linked to redistribution of richness patterns, and maximum temperature anomaly to compositional change. Critically, the regions predicted to experience the most severe declines are also those facing the highest deforestation rates and lowest protected area coverage, meaning that climate change impacts are likely to be compounded by ongoing habitat loss. These findings underscore the urgency of conservation strategies that enhance habitat connectivity and protect climatically stable areas in the most biodiverse and threatened portions of the biome.

## Author Contributions

**Amanda Sabino:** conceptualization (lead), data curation (lead), formal analysis (lead), investigation (lead), methodology (equal), software (equal), validation (equal), visualization (lead), writing – original draft (lead), writing – review and editing (equal). **Gabriela Alves‐Ferreira:** conceptualization (equal), data curation (equal), formal analysis (equal), methodology (equal), software (equal), supervision (equal), validation (equal), visualization (equal), writing – review and editing (equal). **Neander Marcel Heming:** conceptualization (equal), formal analysis (equal), methodology (equal), software (equal), supervision (equal), validation (equal), visualization (equal), writing – review and editing (equal). **Gastón Andrés Fernandez Giné:** formal analysis (equal), methodology (equal), software (equal), validation (equal), visualization (equal), writing – review and editing (equal). **Daniela Custódio Talora:** conceptualization (equal), investigation (equal), methodology (equal), supervision (lead), validation (equal), visualization (equal), writing – review and editing (equal).

## Funding

This work was supported by Fundação de Amparo à Pesquisa do Estado da Bahia (FAPESB) and Idea Wild.

## Conflicts of Interest

The authors declare no conflicts of interest.

## Supporting information


**Online Resource 1:** Species families.

**Online Resource 2:** Filtered occurrence records for 139 species used for SDMs.

**Online Resource 3:** Contribution of the bioclimatic variables.

**Online Resource 4:** Spatial distribution maps of the climatic predictors (anomalies and velocities) for the Cerrado.

**Online Resource 5:** Protocol to make the workflow and parametrization reproducible.

**Online Resource 6:** Species distribution maps overlaid with occurrence records.

**Online Resource 7:** Performance metrics (AUC and Omission Rates) for the ensemble models. models_maxent.R: Script used to build Species Distribution Models.

## Data Availability

All data used in this study, along with the [Link], [Link], [Link], [Link], [Link], [Link], [Link], are available in the Data Dryad Repository: doi: https://doi.org/10.5061/dryad.s1rn8pkgs.
