# Supplementary material for: Impacts of Climate Change on Alpha and Beta Diversity Patterns of Cerrado anurans
Source: Ecol Evol. 2026 Aug 25;16(8):e73983. doi: 10.1002/ece3.73983 (PMC13504677; doi:10.1002/ece3.73983)
Supplement: Supplementary file 1 [file ECE3-16-e73983-s005.pdf]

Climate change effects on alpha and beta diversity patterns of Cerrado anurans

– ODMAP Protocol –

2024-07-30

Overview

Model objective

Model objective: Forecast and transfer

Focal Taxon

Focal Taxon: Using SDMs we constructed potential distribution maps for Cerrado anurans.

Location

Location: Our focal study area is the Cerrado biome, Brazil.

Scale of Analysis

Spatial extent: xmin: -60.4726 ymin: -24.68178 xmax: -41.27754 ymax: -2.332088

Spatial resolution: 2.5 arc minutes (~4.5 km^2 at the equator)

Temporal extent: We used occurrence records collected until 2022.

Temporal resolution: baseline (1970-2000) and future scenario 2050 (2041-2060).

Boundary: natural, Cerrado biome boundary.

Biodiversity data

Observation type: Occurrence records were obtained using the Global Biodiversity Information Facility (GBIF: https://www.gbif.org) and Scientific literature (data provided by Vasconcelos et al., 2018).

Response data type: point occurrence

Predictors

Predictor types: climatic

Hypotheses

Hypotheses: The central hypothesis of your study is that climate change will lead to a reduction in climatically suitable areas for Cerrado anurans, resulting in a decrease in species richness and significant changes in species composition.

Assumptions

Model assumptions: Our assumptions are that species are at equilibrium with their environment, occurrence records are adequate and representative, the ecological niche is preserved over time and our models do not account for the potential of species to adapt to climate change.

Algorithms

Modeling techniques: maxent

Model complexity: We considered all the possible combinations of Feature classes (FCs): linear (L), product (P), quadratic (Q), hinge (H) and Regularization multipliers (RMs) with values between 0.5 and 5.

Model averaging: Consensual models for each species were developed using 10% of of the models with the best performances, which were chosen based on the lowest omission rate (OR) values and the highest area under the curve (AUC) averages, following the sequential procedure suggested by Boria et al. (2017).

Workflow

Model workflow: We used the filtered occurrence data and the selected bioclimatic variables to predict climatically suitable areas for 139 anurans in the Cerrado. We built SDMs through the MaxEnt algorithm (Phillips et al., 2017) using the *ENMwizard* package (Heming et al. 2018) from R software (R Core Team, 2023). We defined the calibration area for model fitting through a 1.5° buffer around a Minimum Convex Polygon (MCP) involving all filtered occurrence points for each species (Anderson & Raza, 2010; Barve et al., 2011; Heming et al., 2018). To apply a model selection structure, we build 70 models for each species, considering all the possible combinations of Feature classes (FCs): linear (L), product (P), quadratic (Q), hinge (H) and Regularization multipliers (RMs) with values ranging between 0.5 and 5, with interval of 0.5. For validation and evaluation of the predictive ability of the models, we applied two methods of geographic partitioning of occurrence records: 'block' and 'jackknife' using the ENMevaluate_b function of the *ENMwizard* package (Heming et al 2018). Block cross-validation partitions the occurrence points into different blocks for model evaluation and calibration (Roberts et al., 2017), while the jackknife method removes only one point at a time and builds a new model in the absence of that point (Pearson, 2010; Shcheglovitova & Anderson, 2013). We used the jackknife method for species with fewer than 15 occurrence records, while the block method was used for species with more than 15 records. We calibrated and evaluated the models with the current baseline climate scenario. After this procedure, we selected 10% of the models with the best performances, which were chosen based on the lowest omission rate (OR) values and the highest area under the curve (AUC) averages, following the

sequential procedure suggested by Boria et al. (2017). We projected the selected models for the baseline and future scenarios, considering the year 2050, one pessimistic shared socioeconomic pathway (SSP585). We calculated the weighted average to generate a consensus model for each species and each scenario, combining the projections resulting from the three GCMs. Finally, the continuous suitability maps were converted into binary maps (1 = presence and 0 = absence) using the 10 percentile training presence (10ptp) threshold, since the threshold value should be adjusted to the prevalence of presence data (Lobo et al., 2008) and it assumes that 10% of records, with smaller values of environmental suitability, will be discarded (Barros et al., 2012; Liu et al., 2005).

Software

Software: Analyses were conducted in R (version 4.4.1) with the R package ENMwizard (Heming et al. 2018).

Code availability:
https://datadryad.org/stash/share/Fuzq6K78eP94qltp57Wjp4_zmHrHSPfLkGaUrsF4mgs

Data availability:
https://datadryad.org/stash/share/Fuzq6K78eP94qltp57Wjp4_zmHrHSPfLkGaUrsF4mgs

Data

Biodiversity data

Taxon names: 139 anuran species from Cerrado.

Taxonomic reference system: We follow the taxonomy of Frost (2023, Darrel Frost and the American Museum of Natural History, Amphibian Species of the World).

Ecological level: communities, species

Data sources: Global Biodiversity Information Facility (http://www.gbif.org, 2023); scientific literature (data provided by Vasconcelos et al., 2018).

<Sampling design>

Sample size: 139 species and 15,601 occurrence records.

Clipping: Cerrado, Brazil

Cleaning: We removed duplicates, incomplete and unlikely coordinates using the scrubr package (Chamberlain, 2021). We also removed records with latitude and longitude coordinates of zero, occurrences located within 2 km of country or capital centroids, occurrences within 2 km of zoos or herbaria, and those located over the ocean. In order to reduce spatial autocorrelation and possible geographic biases, we removed records that were within 10 km of each other using the spThin optimization algorithm (Aiello-Lammens et al., 2015).

Data partitioning

Training data: We applied two methods of geographic partitioning of occurrence records: 'block' and 'jackknife'. Block cross-validation partitions the occurrence points into different blocks for model evaluation and calibration (Roberts et al., 2017), while the jackknife method removes only one point at a time and builds a new model in the absence of that point (Pearson, 2010; Shcheglovitova & Anderson, 2013). We used the jackknife method for species with fewer than 15 occurrence records, while the block method was used for species with more than 15 records.

<Validation data>

Predictor variables

Predictor variables: We only selected bioclimatic variables (those with greater biological relevance) with Pearson's correlation <0.7, resulting in six bioclimatic variables selected as predictors (BIO1, BIO2, BIO15, BIO17, BIO18 and BIO19).

Data sources: WorldClim v2.1 platform (Fick & Hijmans, 2017) https://www.worldclim.org/data/worldclim21.html

Spatial extent: -180, 180, -90, 90 (xmin, xmax, ymin, ymax)

Spatial resolution: 2.5 arc minutes (~4.5 km^2 at the equator)

Coordinate reference system: WGS 1984

Temporal extent: Bioclimatic variables were obtained for the present (1970-2000)

Transfer data

Data sources: WorldClim v2.1 platform (Fick & Hijmans, 2017).

https://www.worldclim.org/data/cmip6/cmip6climate.html

Spatial extent: -180, 180, -90, 90 (xmin, xmax, ymin, ymax)

Spatial resolution: 2.5 arc minutes (~4.5 km^2 at the equator)

Temporal extent: Bioclimatic variables were obtained for the future (2050; 2041-2060)

Models and scenarios: We considered three global circulation models (AOGCMs: CNRM-CM6-1, MIROC6, and MRI-ESM2-0) and two shared socio-economic pathway scenarios, one pessimistic (SSP585) and one optimistic (SSP245).

Model

Variable pre-selection

Variable pre-selection: From a set of 19 bioclimatic variables, we only selected variables (those with greater biological relevance) with Pearson correlation <0.7 to

reduce collinearity problems, resulting in six bioclimatic variables selected as predictors (BIO1, BIO2, BIO15, BIO17, BIO18 and BIO19) to perform the SDMs.

Multicollinearity

Multicollinearity: See “variable pre-selection”

Model settings

maxent: Feature classes (FCs): linear (L), product (P), quadratic (Q), hinge (H) and Regularization multipliers (RMs) with values ranging between 0.5 and 5, with interval of 0.5.

<Model settings (extrapolation)>

Model estimates

<Coefficients>

Model selection - model averaging - ensembles

Model selection: We selected 10% of the models with the best performances, which were chosen based on the lowest omission rate (OR) values and the highest area under the curve (AUC) averages.

Model averaging: We calculated the weighted average to generate a consensus model for each species and each scenario, combining the projections resulting from the three GCMs.

Model ensembles: Consensual models for each species were developed using the top 10% of MaxEnt models, selected based on the lowest Omission Rate (OR) and the highest average Area Under the Curve (AUC) values, following the criteria outlined by Boria et al. (2017).

Threshold selection

Threshold selection: The continuous suitability maps were converted into binary maps (1 = presence and 0 = absence) using the 10 percentile training presence (10ptp) threshold.

Assessment

Performance statistics

We selected 10% of the best performing models with the best performances, which were chosen based on the lowest omission rate (OR) values and the highest area under the curve (AUC) averages, following the sequential procedure suggested by Boria et al. (2017).

Plausibility check

Prediction

Prediction output

Prediction unit: Projections of species richness in the baseline scenario and 2050, and shifts in species composition.

Uncertainty quantification
