# Supplementary material for: Impacts of Climate Change on Alpha and Beta Diversity Patterns of Cerrado anurans
Source: Ecol Evol. 2026 Aug 25;16(8):e73983. doi: 10.1002/ece3.73983 (PMC13504677; doi:10.1002/ece3.73983)
Supplement: Supplementary file 2 [file ECE3-16-e73983-s007.pdf]

Supplementary material S4

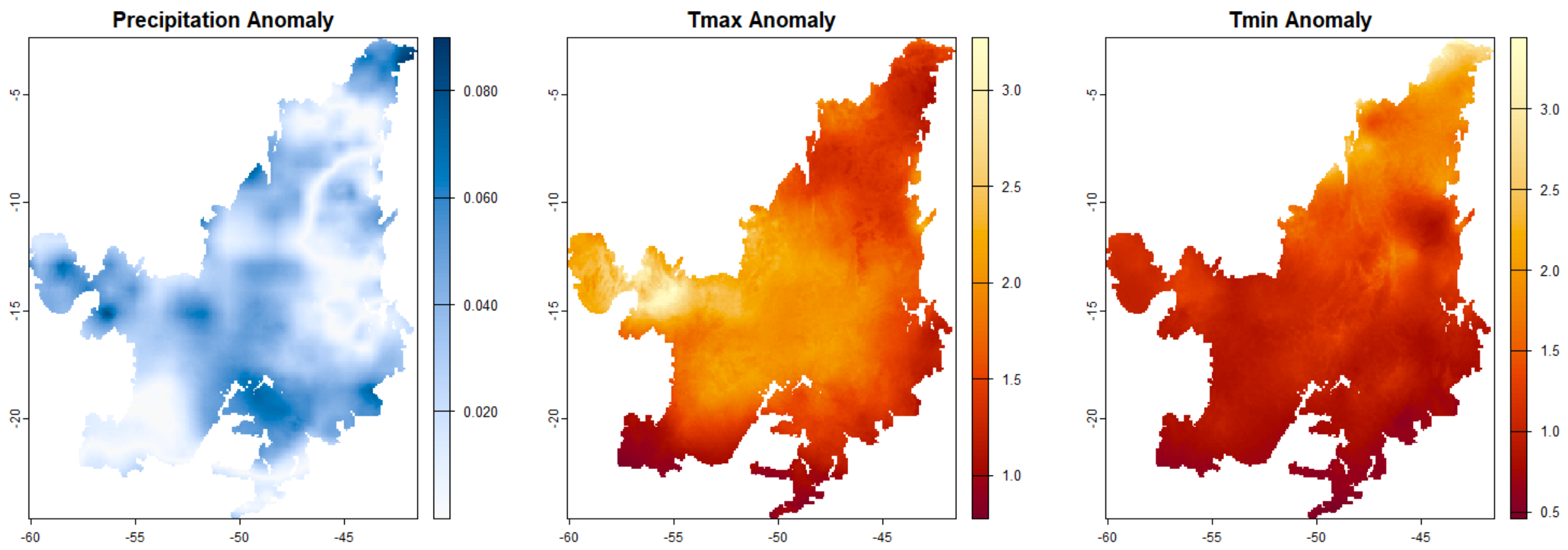

Figure S4.1. Spatial distribution of standardized Euclidean distance (SED) climatic anomalies projected for 2050 under the SSP585 scenario across the Cerrado biome: (a) precipitation anomaly, (b) maximum temperature (Tmax) anomaly, and (c) minimum temperature (Tmin) anomaly. Color scales represent the magnitude of the anomaly for each variable, with darker/warmer tones indicating greater deviation from current climatic conditions.

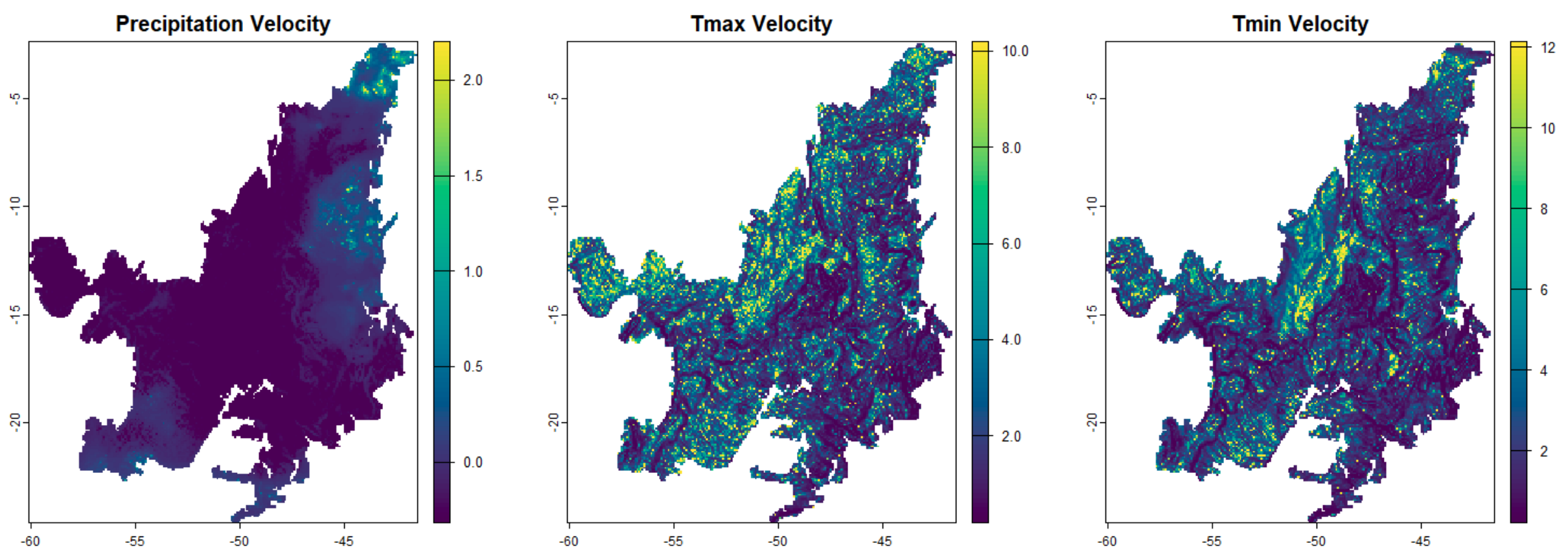

Figure S4.2. Spatial distribution of climate change velocity (km/year) projected for 2050 under the SSP585 scenario across the Cerrado biome: (a) precipitation velocity, (b) maximum temperature (Tmax) velocity, and (c) minimum temperature (Tmin) velocity. Color scales indicate the rate of displacement of climatic conditions across space, with higher values (yellow/green) representing areas of faster climatic change.
